# Cresol salting out and complexation with metal salts influence their migration in impacted aquifers

**DOI:** 10.1007/s43832-026-00402-6

**Published:** 2026-06-16

**Authors:** Renato Souza Lima Sant’Anna, Alejandro G. Marangoni, Erica Pensini

**Affiliations:** 1 Department of Civil, Environmental and Water Engineering, College of Engineering, University of Guelph, 50 Stone Road East, Guelph, ON N1G 2W1 Canada; 2 Biophysics Interdepartmental Group (BIG), University of Guelph, 50 Stone Road East, Guelph, ON N1G 2W1 Canada; 3 Food Science Department, University of Guelph, 50 Stone Road East, Guelph, ON N1G 2W1 Canada

**Keywords:** Cresols (m-cresol, p-cresol), Salting out, Liquid–liquid phase separation (LLPS), Metal–phenol complexation, ATR-FTIR spectroscopy, Droplet transport

## Abstract

Salts and transition metals commonly co-occur with phenolic co-pollutants in impacted aquifers and treatment reactors, yet their combined effects on phenol speciation, transport, and removability remain poorly constrained. Here we examine how chloride and sulfate salts of Na, Zn, Fe, and Cu alter the mixing behavior of m-cresol and p-cresol in water and the downstream consequences for mobility and filtration. Bottle tests and optical microscopy show that 0.1–0.5 M electrolytes decrease cresol miscibility and generate polydisperse cresol-rich droplets. Conductor-like Screening Model for Realistic Solvation (COSMO-RS) calculations support salt-driven increases in cresol activity consistent with salting out. Beyond phase separation, metal identity and counter-ion strongly influence cresol association: attenuated total reflectance-Fourier transform infrared spectroscopy (ATR-FTIR) reveals isomer-dependent new bands (815 cm⁻¹ for m-cresol; 775 cm⁻¹ for p-cresol) in selected systems, indicating formation of salt- and isomer-specific cresol–metal species, particularly in chloride media, while sulfate salts suppress or alter these signatures. Density-based “perfect cresol-water separation” predictions indicate that most divalent brines at 0.5 M should buoy cresol. However, observed flotation/sinking deviates systematically where metal association and iron (oxyhydr)oxide floc formation promote droplet densification and entrainment. Using Stokes–Einstein scaling and a geometric straining model, we show that salt-induced droplets shift cresol transport from purely molecular diffusion to size-dependent particle mobility, with multi-micron droplets increasingly susceptible to pore-throat exclusion in finer media. Collectively, these results link electrolyte-driven demixing and complexation to cresol migration pathways, which should inform the management of impacted sites.

**Supplementary Information:**

The online version contains supplementary material available at 10.1007/s43832-026-00402-6.

## Introduction

The detection, transport, and removal of organic pollutants in aqueous environments are often treated as problems of dissolved solutes moving through water. In many impacted systems, however, dissolved inorganic salts and metal ions can fundamentally change pollutant speciation and physical state, shifting contaminants from a “free” molecular form to salt-desolvated clusters, phase-separated droplets, and/or metal-associated species. These transformations can invalidate assumptions embedded in common monitoring and modeling approaches, because both analytical signatures (e.g., vibrational spectra) and transport controls (e.g., buoyancy, straining, filtration capture) become functions of water chemistry rather than intrinsic properties of the organic molecule alone.

Cresols (methylphenols), including m-cresol and p-cresol, are among the most prevalent industrial phenolic pollutants. They are used as chemical intermediates and solvents, in pesticide synthesis, wood preservation, and petrochemical and coal-processing operations [1–3], and they occur in phenolic waste streams from coal coking and gasification [4–7]. In coke/coking (coal) wastewaters and related legacy-impacted receiving waters, cresols are common phenolic constituents, and these matrices often exhibit high inorganic-salt loads (e.g., chloride/sulfate) and measurable transition metals including Fe, Zn, and Cu [4]. This co-occurrence reflects common source pathways: coal processing can generate phenolic effluents while iron and zinc enter solution via catalysts and infrastructure; electroplating operations employ zinc and copper salts and may introduce cresols as process additives or degreasing agents; and agrochemical manufacturing can involve Cu/Fe/Zn catalysts during synthesis and purification of cresol-derived herbicides and related intermediates. The resulting mixed organic–inorganic matrices pose a dual challenge: salts can reduce organic solubility (“salting out”) [8], and metal ions can engage in coordination chemistry with phenolic functional groups [9, 10], with consequences for both contaminant characterization and fate.

From an analytical perspective, coordination chemistry is particularly important because metal–phenol interactions can alter the spectroscopic “fingerprint” used to detect and quantify phenols. The classic ferric-chloride response of phenols illustrates how Fe(III)–phenolate chromophores can produce strong visible signals [10]; in complex matrices, analogous association can generate new diagnostic features or suppress expected ones. For cresols, isomeric geometry adds an additional dimension: m-cresol and p-cresol present the same functional group but differ in substitution pattern, which can influence the propensity to form metal-associated species under a given anion environment (chloride versus sulfate). Understanding when such interactions occur—and how they manifest spectroscopically—is essential for interpreting IR measurements in real waters where salts and metals may shift band positions, create new bands, or change phase partitioning in ways that bias sampling and analysis.

From a transport and remediation perspective, salt-driven phase separation can transform cresols from dissolved solutes into a dispersed droplet phase with size-dependent mobility. In groundwater and treatment reactors, the transition from dissolved transport (governed by molecular diffusion and advection–dispersion) to droplet or cluster transport introduces controls more commonly associated with multiphase or colloid-like systems [11, 12]: buoyancy versus settling, aggregation with metal (oxyhydr)oxide flocs, pore-throat straining, and filtration capture by interception/coalescence. These processes can reorganize plume architecture (e.g., upward accumulation beneath low-permeability layers versus downward migration as droplet–floc aggregates), change residence times, and create persistent secondary sources. They also imply that treatment approaches effective for dissolved cresols may be ineffective once cresols are present as droplets—and vice versa—highlighting the need for purification strategies that explicitly account for physical state and wetting behavior.

Key knowledge gaps remain at the intersection of electrolyte-driven phase behavior and transition-metal chemistry for cresols. Specifically, (i) the electrolyte concentrations and counter-ions that drive cresol demixing under water-relevant conditions are not commonly connected to measurable droplet properties (size distributions, buoyancy, and mobility); (ii) the extent to which cresol isomer structure controls metal association in chloride versus sulfate media—and how such association perturbs ATR-FTIR signatures used for monitoring—has not been systematically linked to phase-separated behavior; and (iii) the practical implications of these coupled effects for subsurface transport (including straining and floc-mediated retention) and for simple, scalable removal strategies are not well constrained. Addressing these gaps is necessary to interpret cresol measurements in complex waters and to select treatment approaches that remain effective across dissolved, droplet, and metal-associated regimes.

**Scheme 1:**
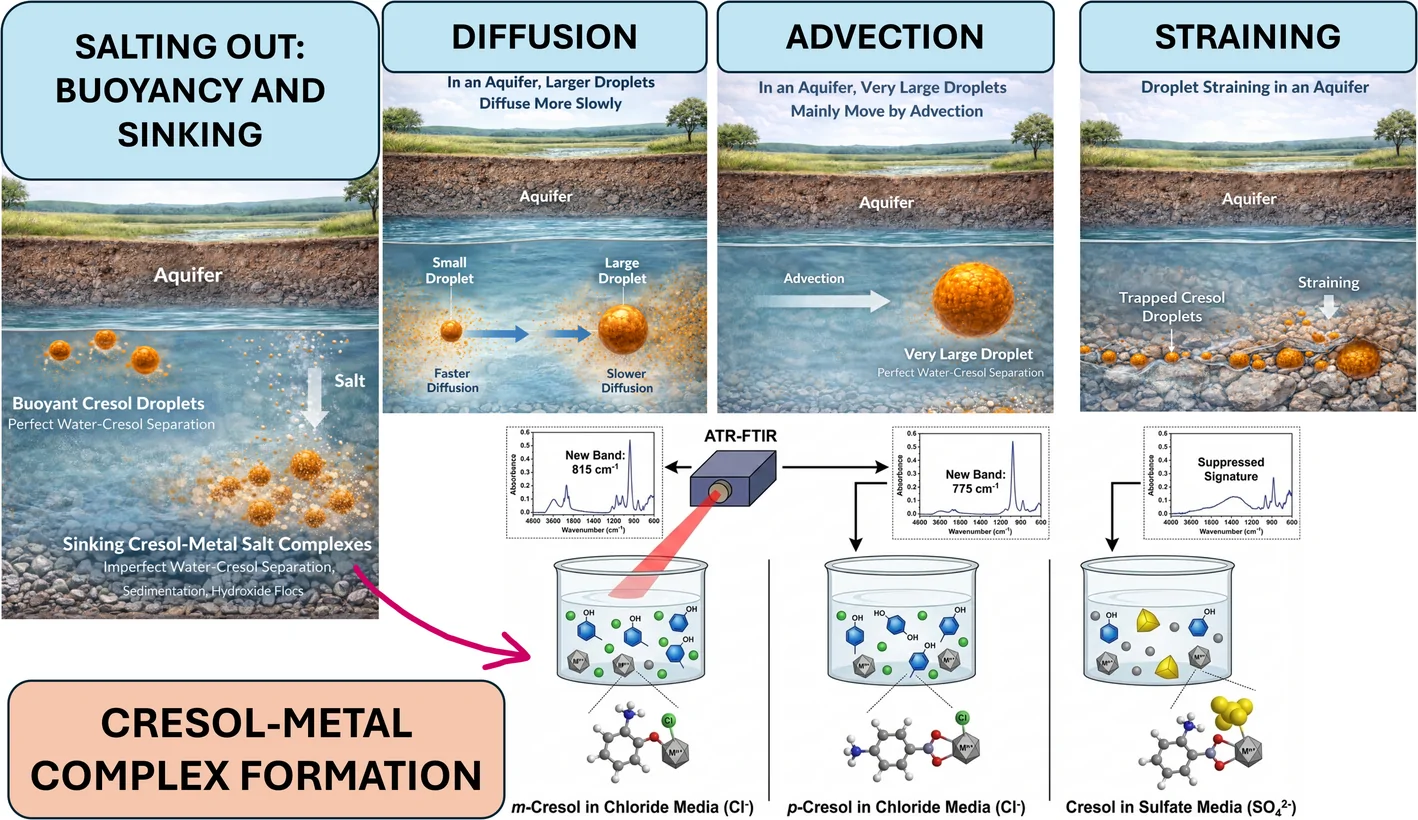
Conceptual schematic illustrating first-order transport regimes expected under electrolyte- and metal-controlled cresol demixing, transport, and filtration behavior. Electrolytes (0.1–0.5 M) “salt out” cresols from water, producing polydisperse cresol-rich droplets whose density governs buoyant rise or sinking. As droplet size increases, transport shifts from rapid molecular diffusion (small droplets/solute) to slower size-dependent spreading and ultimately advection-dominated motion for very large droplets. In porous media, multi-micron droplets become susceptible to geometric straining and pore-throat exclusion, leading to droplet trapping. ATR-FTIR signatures indicate concurrent, ion-specific association: chloride salts can promote isomer-dependent cresol–metal species (new bands near 815 cm⁻¹ for m-cresol and 775 cm⁻¹ for p-cresol), whereas sulfate salts suppress or alters these complexation signatures. Metal association and iron (oxyhydr)oxide floc formation can densify/entrain droplets, yielding deviations from “perfect” buoyancy-based water–cresol separation

In this study, we investigate how salt identity, counter-ion (chloride vs. sulfate), and cresol isomer jointly control (i) salting out and droplet formation, (ii) salt- and isomer-dependent cresol–metal association detectable by ATR-FTIR, (iii) buoyancy and mobility of separated droplets relative to density-only “perfect separation” predictions, and (iv) practical removal by filtration. Bottle tests and optical microscopy are used to document demixing and droplet polydispersity across Na, Zn, Fe, and Cu chloride/sulfate systems. ATR-FTIR is used to probe association, with particular attention to isomer-dependent bands observed in selected metal-salt systems (e.g., an 815 cm^−1^ marker in m-cresol systems and a 775 cm^−1^ marker in p-cresol systems). We then connect these molecular-scale signatures to macroscopic behavior via simple density-based flotation predictions, Stokes–Einstein scaling for droplet diffusion, and a geometric straining framework. These transport analyses are intended as first-order conceptual/scaling tools to identify likely transport regimes and governing physical controls under salt-induced phase separation, rather than as full predictive transport models for heterogeneous aquifers. Finally, we discuss water purification strategies that exploit the phase change and, where cresols remain dissolved, polymer-assisted binding (using polyvinyl pyrrolidone, PVP) to render cresols filter-retainable. PVP is selected because of its known association with phenolic and polyphenolic compounds [13–16]. By linking coordination chemistry, electrolyte-driven phase behavior, and separations mechanics, this work provides an integrated basis for interpreting cresol signatures in complex waters and for selecting treatment approaches consistent with the pollutant’s operative physical state, as illustrated in Scheme 1.

## Materials and methods

### Materials

p-Cresol (99%), m-Cresol (99%), FeCl_2_·4H_2_O (> 99% pure), FeSO_4_·7H_2_O (> 99% pure), Na_2_SO_4_ (> 99% pure), and polyvinylpyrrolidone (PVP) were purchased from Sigma Aldrich (Canada), Copper (II) Sulfate pentahydrate (≥ 98%), Copper (II) chloride dihydrate (99%) from Fisher Scientific Canada. Syringe Filters 33 mm diameter, 0.22 μm pore size, Mixed Cellulose Ester (MCE), and 25 mm diameter, 0.2 μm Nylon, both sterile and hydrophilic, were purchased from Fisher Scientific (Canada). All experiments were conducted using Milli-Q water (18.2 MΩ cm).

### Sample preparation and treatment

Different sets of samples were prepared and treated using different approaches, as outlined below and as summarized in Table 1. All samples were prepared, stored and analyzed under ambient conditions (ambient pressure and atmosphere, and room temperature ≈ 19–20 °C). Accordingly, iron-containing systems should be viewed as open systems in which partial oxidation/hydrolysis of Fe(II) may occur during aging.

- *SET 1*. Aqueous mixtures (0.1 and 0.5 M) of NaCl or metal salts (FeCl_2_, FeSO_4_, CuCl_2_, CuSO_4_, ZnCl_2_, ZnSO_4_) and 2 wt% m-Cresol or p-Cresol. The 2 wt% concentration of m-cresol and p-cresol was selected because it is below the solubility of these compounds in water at room temperature. At that concentration, no separation or droplets are observed with the naked eye or optical microscopy with either p-cresol or m-cresol in the absence of salts. Samples were prepared in glass vials and mixed by hand for 30 s, by inverting the vials, at room temperature (20.5 °C) and ambient pressure. Samples were observed over time, over a period of up to 10 days, to determine their mixing behaviour in water. They were further analyzed by optical microscopy (Sect.  2.4) and ATR-FTIR (Sect.  2.3).
- *SET 2*. Samples were prepared with 2 wt% p-cresol or m-cresol added in water, in the absence of salts or with 0.5 M salts (FeCl_2_, FeSO_4_, CuCl_2_, CuSO_4_). After mixing for 30 s by vial inversion, 1 wt% PVP (relative to the total weight of the mixture) was added and vials were further agitated for 30 s. After 1 h, samples were centrifuged for 3 min and the aqueous phase and the deposit were tested by ATR-FTIR (cf. Section  2.3). Moreover, additional samples were prepared following the same procedure, and after centrifugation, the aqueous phase was filtered using MCE and Nylon (0.22 μm and 0.2 μm, respectively) syringe filters and tested by ATR-FTIR.

The pH of the samples was measured using pH indicator strips at the start of the tests and at defined aging times (5 and 10 days) to assess solution acidity.

**Table 1:** Summary of experiments conducted using samples prepared with m-cresol and p-cresol, with or without salts, and with or without PVP

Sample composition	Aged/equilibrated	Observations	Purpose of observations
m-cresol and p-cresol: 2 wt% SALTS: NaCl, FeCl_2_, FeSO_4_, CuCl_2_, CuSO_4_, ZnCl_2_, ZnSO_4_ (0.1 M and 0.5 M) Samples without salts were analyzed for comparison	Up to 10 days	Visual observations, optical microscopy and ATR-FTIR	Mixing behaviour, buoyancy and cresol-salt complex formation
m-cresol and p-cresol: 2 wt% SALTS: none PVP: 1 wt%	1 h	ATR-FTIR after centrifugation and filtration	Removal of cresols from water
m-cresol and p-cresol: 2 wt% SALTS: 0.5 M of either FeCl_2_, FeSO_4_, CuCl_2_ or CuSO_4_ PVP: 1 wt%	1 h	ATR-FTIR after centrifugation	Removal of cresols from water

### ATR-FTIR

ATR-FTIR measurements were conducted to analyze the following: (1) the formation of complexes between m-cresol and p-cresol, and metal salts. To this end, both the water-rich phase and the separated droplets were collected separately and analyzed; (2) the decrease in m-cresol and p-cresol in water following filtration using MCE or nylon filters.

Absorbance spectra were collected using an ATR-FTIR spectrometer (Thermoscientific Nicolet Summit FTIR spectrometer with an Everest ATR), with an accompanying IR solution software. Each spectrum is the average of 20 scans, with a resolution of 2 cm^−1^, in the wavenumber range of 400 cm^−1^ to 4000 cm^−1^. All experiments were conducted in triplicate at least. Spectra were analyzed using Quasar v. 1.5.0 (orange spectroscopy). Baseline corrections were done with a rubber band baseline type spectra were normalized using min/max normalization. A calibration curve was used to quantify m-cresol and p-cresol removal from water. This calibration curve was obtained using m-cresol and p-cresol in water standards (Additional file 1: Fig. S1). Because the 1500–1550 cm^−1^ band of m-cresol and p-cresol partially overlaps the water δ(HOH) band, we used an empirical area-ratio metric: the sum of the integrated area of the m-cresol and p-cresol bands at 1200–1300 cm^−1^ and 1415–1530 cm^−1^ was divided by the integrated area over 1200–1900 cm^−1^, a window dominated by the water δ(HOH) band but also containing m-cresol and p-cresol contributions. This ratio showed a monotonic dependence on m-cresol and p-cresol concentration. Over 0.5–2 wt% m-cresol and p-cresol in water, the response was reasonably described by a linear fit (R^2^ =0.9865 and R^2^ = 0.9896 for p-cresol and m-cresol, respectively), enabling semi-quantitative tracking of m-cresol and p-cresol depletion.

### Optical microscopy

A Keyence VHX-5000 digital microscope (Keyence Corporation, Canada) was used to image samples prepared as described in Sect.  2.2 to observe the formation of droplets and metal hydroxide flocs.

### Modelling with COSMO-RS (COnductor-like Screening MOdel for Realistic Solvents)

COSMO-RS was used as a complementary thermodynamic screening tool to estimate activity coefficients of aqueous mixtures containing m-cresol and NaCl. To enforce electroneutrality, Na^+^ was included in aqueous mixtures containing the chloride anion (with equimolar ratios of Na^+^ and Cl^−^).

- *Molecular model construction and COSMO file generation (AMS/ADF)*. Quantum-chemical structure preparation and COSMO surface generation were performed in Amsterdam Modeling Suite (AMS) 2025.102 using AMSinput 2025.102 (ADF engine). Molecular structures were prepared for the solutes required in COSMO-RS analyses, distinguishing between (i) species taken directly from the COSMO-RS compound database and (ii) species constructed explicitly in AMSinput.
- *Species definitions and provenance*. Neutral m-cresol and water, as well as the chloride anion were taken directly from the COSMO-RS database. An additional Na^+^ species was generated in-house in AMSinput.
- *Geometry optimization settings*. All in-house species were subjected to gas-phase geometry optimization with COSMO enabled (see below). Calculations were run as Geometry Optimization jobs (no vibrational frequency calculations). The following ADF settings were used: XC functional = LDA, basis set = DZ, frozen core = Large, numerical quality = Normal, and relativity = Scalar. All calculations were performed as restricted closed-shell (unrestricted disabled), as the studied species are closed-shell organic ions/ion pairs. Formal charge was set in the ADF “Main” panel to enforce the intended speciation. The total charge of Na⁺ was set to + 1.
- *COSMO solvation model settings for COSMO-RS compatibility*. COSMO files were generated using the ADF COSMO implementation under Model → Solvation → COSMO, with COSMO solvent = CRS (COSMO-RS compatible setting) and default radii = Klamt. The optimized structure was exported as the COSMO-RS input species (e.g., *.coskf).

The parameters and settings used in COSMO-RS calculations are provided in the supporting information file Additional file 1: Fig. S2).

### Density-based flotation threshold and rise/sink velocity calculations

To provide a first-order conceptual/scaling estimate of buoyancy-driven behavior after phase separation, the buoyancy of cresol-rich droplets in electrolyte solutions was evaluated using a density-only framework assuming complete phase separation (droplet phase: pure m-cresol or p-cresol; continuous phase: aqueous electrolyte), while neglecting complexation, entrained solids/flocs, and droplet entrainment by flocculation.

At a given temperature $\:\mathrm{t}$(°C), the density of the aqueous electrolyte solution, $\:{{\uprho\:}}_{\mathrm{b}\mathrm{r}\mathrm{i}\mathrm{n}\mathrm{e}}$, was estimated using the Laliberté-Cooper semi-empirical density model for single-electrolyte aqueous solutions [17]. Because the full implementation requires auxiliary relations for pure-water density, electrolyte apparent specific volume, and conversion between molality and molarity, these details are provided in Section S3 of the Supporting Information.

The limiting (turning-point) salt concentration for flotation, $\:{\mathrm{M}}^{\mathrm{*}}$, was defined by the condition $\:{{\uprho\:}}_{\mathrm{b}\mathrm{r}\mathrm{i}\mathrm{n}\mathrm{e}}\left({\mathrm{M}}^{\mathrm{*}}\right)={{\uprho\:}}_{\mathrm{d}\mathrm{r}\mathrm{o}\mathrm{p}}$, where $\:{{\uprho\:}}_{\mathrm{d}\mathrm{r}\mathrm{o}\mathrm{p}}$ is the density of pure cresol (taken as $\:{{\uprho\:}}_{\mathrm{m}\mathrm{-cresol}}$= 1.034 g mL^−1^ and $\:{{\uprho\:}}_{\mathrm{p}\mathrm{-cresol}}$= 1.0341 g mL^−1^ at 20 °C). For $\:\mathrm{M}<{\mathrm{M}}^{\mathrm{*}}$, droplets were predicted to sink; for $\:\mathrm{M}>{\mathrm{M}}^{\mathrm{*}}$, droplets were predicted to float.

Rise/sink velocities were estimated using Stokes’ terminal velocity for a spherical droplet in a Newtonian continuous phase:

1 $$v=(2r^2 g({{\uprho\:}}_{brine}-{{\uprho\:}}_{drop} ))/9\:{\upmu\:}$$

where $\:\mathrm{r}$ is droplet radius (m), $\:\mathrm{g}$ is gravitational acceleration (9.80665 m s^−2^), and $\:{\upmu\:}$ is the dynamic viscosity of the continuous phase (Pa·s). Positive $\:\mathrm{v}$ indicates upward motion (flotation), whereas negative $\:\mathrm{v}$ indicates downward motion (sedimentation). The characteristic transit time across a liquid height $\:\mathrm{H}$was calculated as $\:{\mathrm{t}}_{\mathrm{t}\mathrm{r}\mathrm{a}\mathrm{n}\mathrm{s}\mathrm{i}\mathrm{t}}=\mathrm{H}/\mid\:\mathrm{v}\mid\:$, with $\:\mathrm{H}$ = 2 cm in this study.

### Diffusion coefficient calculations

To provide order-of-magnitude estimates for likely transport regimes, diffusion coefficients for cresols were evaluated for two idealized cases: (i) molecular diffusion of dissolved cresol below its solubility limit (single-phase aqueous transport) and (ii) Brownian diffusion of cresol-rich droplets/clusters following phase separation (two-phase transport). These calculations are intended as first-order scaling estimates rather than predictive descriptions of aquifer-scale mass transport. Unless otherwise stated, estimates were evaluated at 20 °C using water as the continuous phase.

For dilute aqueous solutions below solubility, the diffusion coefficient of dissolved cresol in water, $\:{\mathrm{D}}_{\mathrm{a}\mathrm{q}}$, was assigned a representative small-organic value of approximately 0.8 × 10^−9^ m^2^ s^−1^. This value is within the typical range for neutral organic solutes of comparable molecular size in water at room temperature and was used identically for m-cresol and p-cresol for order-of-magnitude comparison.

Following phase separation, cresol-rich entities were treated as spherical droplets or clusters of radius $\:\mathrm{a}$. Their translational diffusion coefficient, $\:{\mathrm{D}}_{\mathrm{p}}\left(\mathrm{a}\right)$, was estimated using the Stokes–Einstein relation:

2 $$\:\begin{array}{cccc}&\:{\mathrm{D}}_{\mathrm{p}}\left(\mathrm{a}\right)=\frac{{\mathrm{k}}_{\mathrm{B}}\mathrm{T}}{6{\uppi\:}{\upmu\:}\mathrm{a}}&\:&\:\end{array}$$

where $\:\mathrm{a}$ is droplet radius (m), $\:\mathrm{T}$ is absolute temperature (K), $\:{\mathrm{k}}_{\mathrm{B}}$ is Boltzmann’s constant, and $\:{\upmu\:}$ is the dynamic viscosity of the continuous phase (Pa·s). For reporting, droplet sizes spanning the experimentally observed polydisperse range were evaluated directly from this expression.

For both regimes, a characteristic diffusive spreading length over time $\:\mathrm{t}$was estimated from the one-dimensional diffusion scaling,

3 $$\:\begin{array}{cccc}&\:\mathcal{l}\left(\mathrm{t}\right)\sim\:\sqrt{2\mathrm{D}\mathrm{t}}&\:&\:\end{array}$$

where $\:\mathrm{D}$ is $\:{\mathrm{D}}_{\mathrm{a}\mathrm{q}}$ for dissolved cresol or $\:{\mathrm{D}}_{\mathrm{p}}\left(\mathrm{a}\right)$ for droplets/clusters.

Additional assumptions used for the diffusion estimates are provided in Section S4 of the Supporting Information.

### Geometric straining model

Potential screening of cresol-rich droplets in porous media was evaluated using a first-order geometric straining framework intended to illustrate likely size-exclusion behavior, while neglecting adhesion/attachment to grains, chemical interactions, capillary effects, droplet deformability, and droplet–floc entrainment.

Droplets were treated as rigid spheres transported through a granular medium characterized by grain size and porosity. A representative pore-throat size was estimated using a simple packing-based scaling relation, with full details provided in Section S5 of the Supporting Information. Under pure straining control, droplets were classified as likely to be strained when their diameter exceeded the characteristic throat diameter,

4 $$d_p\,{\gtrsim}\,2r_{t}$$

where $\:{\mathrm{d}}_{\mathrm{p}}$ is the droplet diameter and $\:{\mathrm{r}}_{\mathrm{t}}$ is the characteristic pore-throat radius. Droplets with $\:{\mathrm{d}}_{\mathrm{p}}\ll\:2{\mathrm{r}}_{\mathrm{t}}$ were considered likely to pass geometric constrictions. This approach provides a conservative first-pass size criterion for droplet immobilization by pore-throat exclusion in porous media of different textures and porosities.

## Results and discussion

### Mixing behaviour of m-cresol and p-cresol in water in the presence of salts

**Fig. 1:**
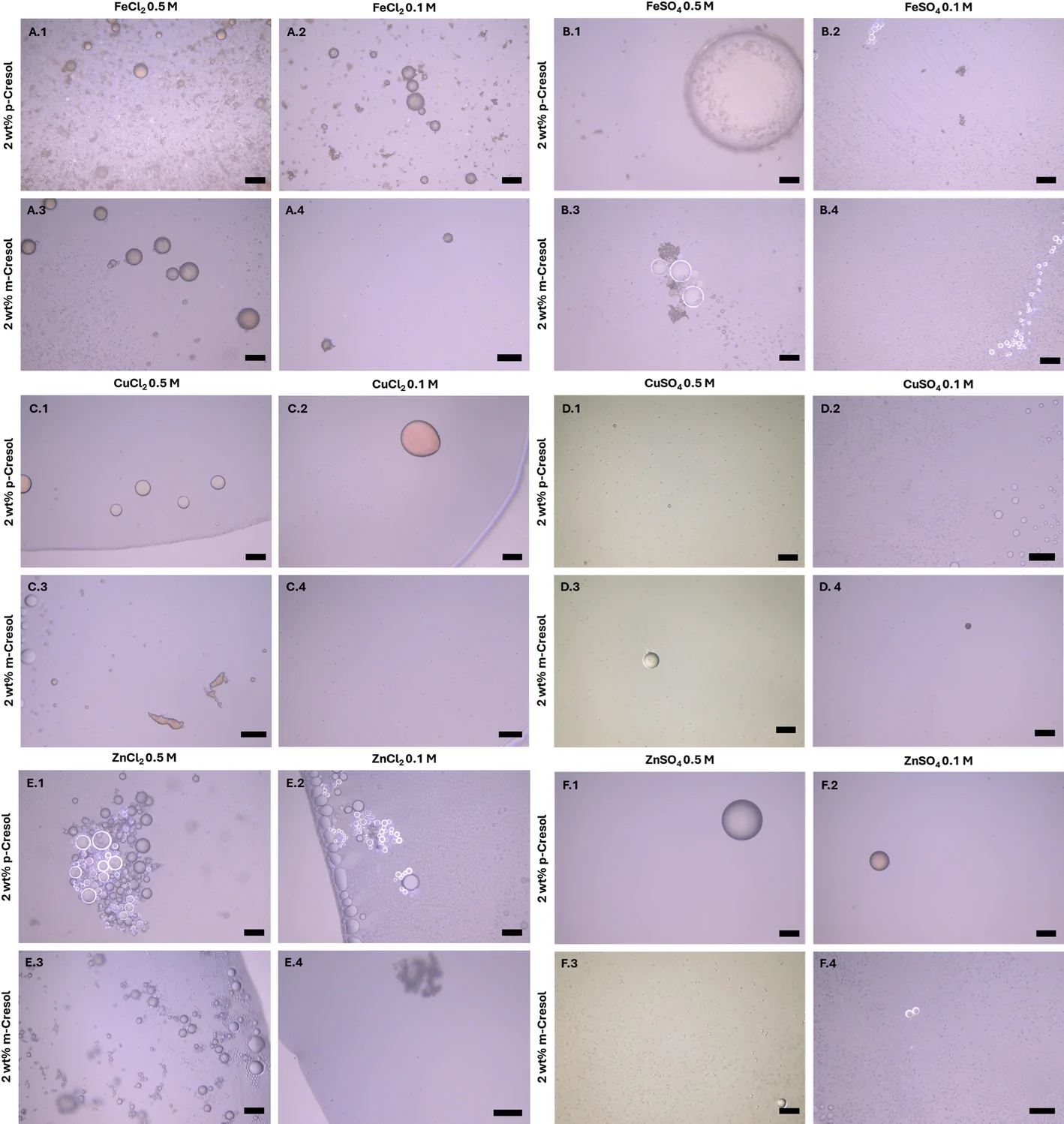
Optical microscopy images of aqueous mixtures (0.1 M and 0.5 M) of metal salts (Panel A: FeCl_2_, B: FeSO_4_, C: CuCl_2_, D: CuSO_4_, E: ZnCl_2_, F: ZnSO_4_) and 2 wt% m-Cresol or p-Cresol. The scale bar is 100 μm. Droplet size and distribution varied across all conditions. Floc aggregation was observed in the FeCl_2_ samples (Panel A) and FeSO_4_ samples imaged (B.1, B.2, B.3). ZnCl_2_ samples (images E.1–3) show aggregated droplets with variable sizes. Note that the edges of the samples are visible in C.1, C.2, and E.2–4

At 20 °C, the reported miscibility limits of m-cresol and p-cresol in pure water are 2.2–2.4 wt% and 1.9–2.3 wt%, respectively [18, 19]. Salts (NaCl, CuCl_2_, CuSO_4_, FeCl_2_, FeSO_4_, ZnCl_2_, and ZnSO_4_) were added to mixtures containing water and cresols. Across the salt-containing systems, qualitative pH measurements made at the start of the experiments and after aging indicated that the samples remained mildly acidic overall (typically ~ 4–6), with no large bulk pH shifts over 5–10 days. Salts decreased the miscibility of both m-cresol and p-cresol in water, leading to droplet formation observed by optical microscopy at salt concentrations of 0.1 M and 0.5 M (Fig. 1 and Additional file 1: Figs. S3–S8, Supporting Information). Larger droplets were also observed with the naked eye. Optical microscopy confirmed the presence of micron-scale and larger droplets, but the full sub-micron droplet-size distribution was not quantified in this study. Photographs of the mixtures (bottle tests) are also provided in the Supporting Information file (Additional file 1: Figs. S9–S14). Droplet formation provides a direct physical basis for altered transport and treatment behavior, because cresol can partition into an organic-rich dispersed phase whose buoyancy, mobility, and straining behavior differ from those of the dissolved solute.

To provide a thermodynamic perspective on the salting-out trend, COSMO-RS calculations were conducted for binary mixtures of m-cresol and water at m-cresol mole fractions of 1 × 10^−5^–5 × 10^−5^. Under these conditions, the activity coefficients were γ(water) ≈ 1.6 and γ(m-cresol) ≈ 1, indicating positive deviation from ideal mixing. Water activity was calculated as $\:{\mathrm{a}}_{\mathrm{w}}={{\upgamma\:}}_{\mathrm{w}}{\mathrm{x}}_{\mathrm{w}}$; accordingly, $\:{\mathrm{a}}_{\mathrm{w}}$ remained close to unity in these binary mixtures because $\:{\mathrm{x}}_{\mathrm{w}}\approx\:1$ at such dilutions. When electro-neutral mixtures containing equal mole fractions of Na^+^ and Cl^−^ (x(Na^+^)=x(Cl^−^)=0.002) were analyzed at the same m-cresol dilution, $\:{\mathrm{a}}_{\mathrm{w}}$ decreased to ≈ 0.997, whereas γ(m-cresol) increased to > 200. We interpret the large increase in γ(m-cresol) as a strong reduction in the effective solubility of m-cresol in the saline aqueous phase and as consistent with approaching the onset of liquid–liquid separation under saline conditions, in agreement with the experimentally observed salting out. Here, NaCl was used as a representative electrolyte to illustrate the salting-out mechanism thermodynamically; metal salts are treated explicitly in later sections where complexation signatures are observed.

Reduction of the effective water solubility of an otherwise water-miscible organic solvent in the presence of salts (salting out) has been widely reported [8, 20, 21]. Strongly hydrated ions bind water in structured hydration shells, lowering water’s capacity to solvate polar organic molecules and thereby increasing the solute activity in the aqueous phase [8, 20, 21]. The result is decreased solubility and, at sufficiently high salinity for some systems, liquid–liquid phase separation into a salt-rich aqueous phase and an organic-rich phase.

The salt concentrations examined here (0.1–0.5 M; ≈ 6,000–29,000 mg/L as NaCl-equivalent Total Dissolved Solids TDS) fall within brackish-to-saline conditions commonly discussed for groundwater resources (e.g., 3,000–10,000 mg/L as brackish) and remain below many oil- and gas-associated formation/produced waters, which are frequently tens to hundreds of g/L TDS [22–29]. While salting out provides a consistent baseline explanation for droplet formation in our system, the magnitude and character of the response differed across cations/anions and between m-cresol and p-cresol. These contrasts are discussed in the following sections.

### Complex formation between salts and cresols

Separated m- and p-cresol droplets exhibited a slight yellow discoloration typical of cresols in mixtures containing NaCl or zinc salts. In contrast, both the color of the separated cresol-rich droplets and the appearance of the continuous water-rich phase depended strongly on the salt identity (Table 2 and Additional file 1: Figs. S9–S14). Two observations are particularly diagnostic: (i) the purple hue of the water-rich phase in FeCl_2_ solutions, and (ii) the dark-brown discoloration of the m- or p-cresol droplets in systems containing FeCl_2_, FeSO_4_, or CuCl_2_, but not CuSO_4_ or Na_2_SO_4_. These characteristic color changes are consistent with salt-specific chemical interactions, including cresol association with evolving Fe-bearing species and/or aqueous metal speciation changes, which we evaluate further using ATR-FTIR spectroscopy.

**Fig. 2:**
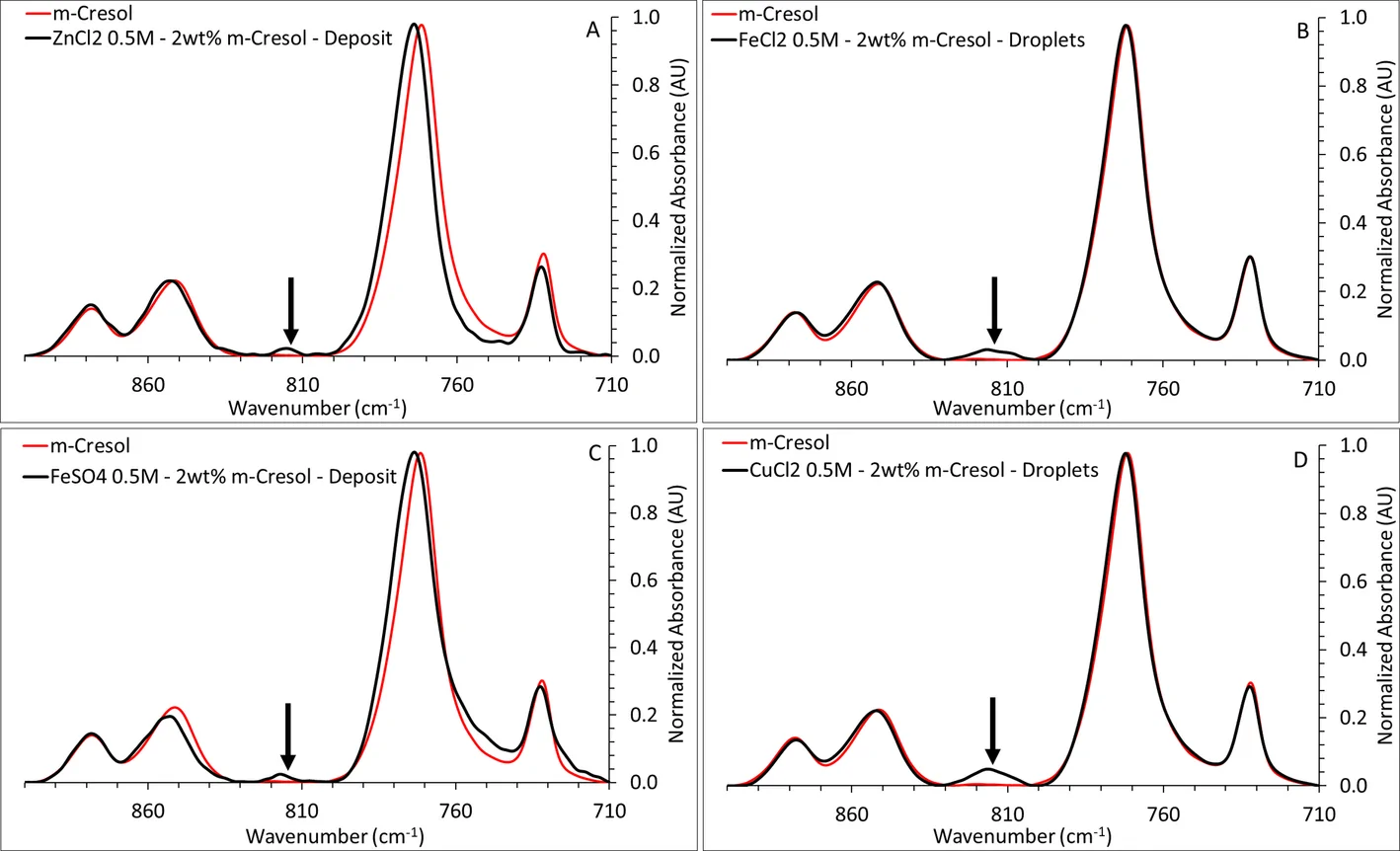
ATR-FTIR spectra of m-cresol droplets separated from systems containing 2 wt% m-cresol and 0.5 M aqueous ZnCl_2_ (**A**), FeCl_2_ (**B**), FeSO_4_ (**C**) and CuCl_2_ (**D**). The panels show the absorbance spectra of m-Cresol and separated or deposited droplets from mixture of 2 wt% m-cresol and 0.5 M aqueous metal salts between 710–900 cm^−1^, as probed by ATR-FTIR. The spectral region of the separated droplets reveals the new peak at 815 cm^−1^, and the characteristic m-cresol strong peak at 775 cm^−1^, assigned to the out-of-plane C–H wagging vibration [30]. The arrows point to the new peak

**Fig. 3:**
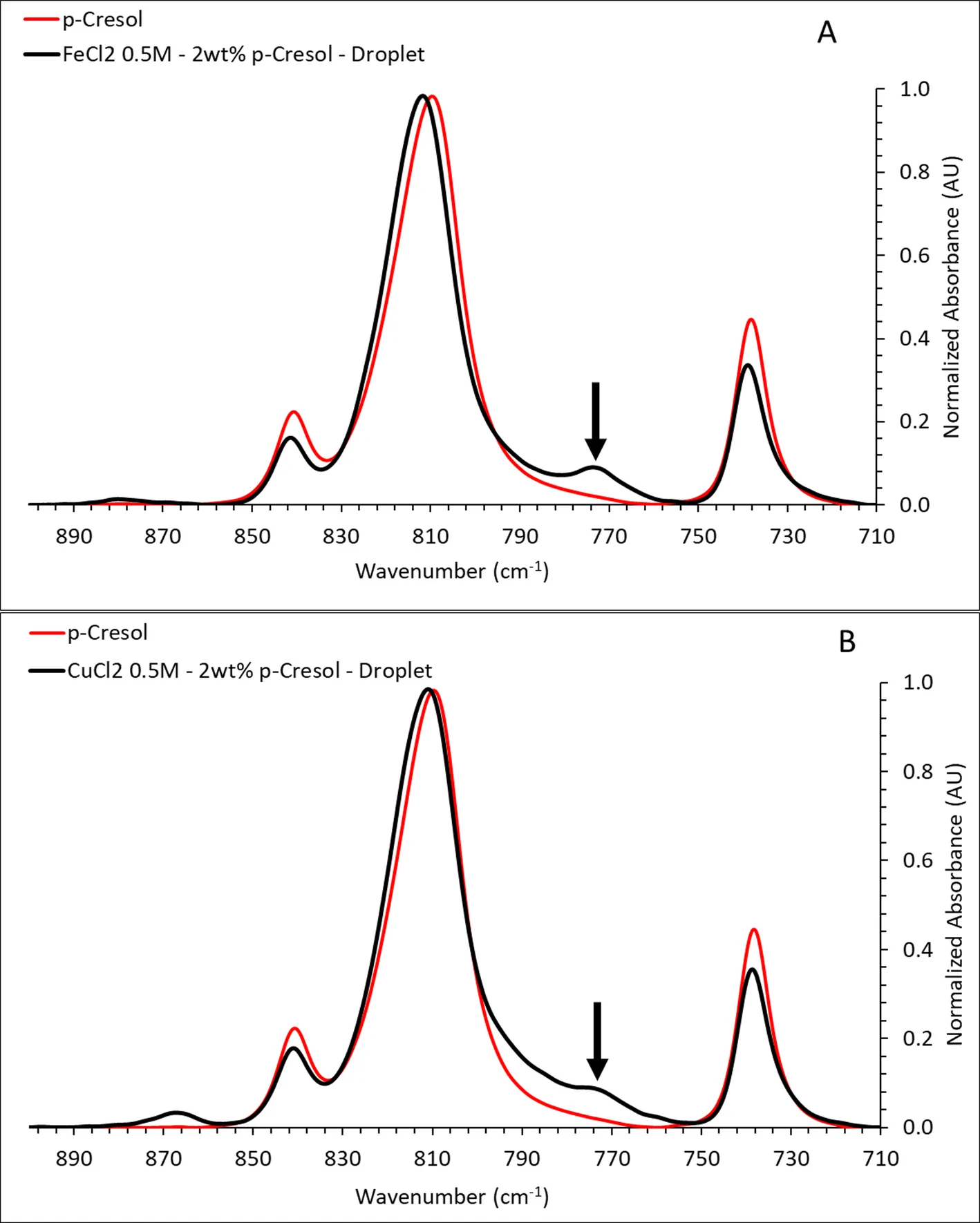
ATR-FTIR spectra of p-cresol droplets separated from systems containing 2 wt% p-cresol and 0.5 M aqueous FeCl_2_ (A) and CuCl_2_ (B). The panels show the absorbance spectra of p-Cresol and separated droplets from mixture of 2 wt% p-cresol and 0.5 M aqueous metal salts between 710–900 cm^−1^, as probed by ATR-FTIR. The separated droplets exhibited a new peak at 775 cm^−1^, while the neat p-cresol characteristic absorption band is at 817 cm^−1^ (CH out of plane wagging, [30]). The new peak is marked by an arrow

ATR-FTIR measurements were conducted to probe whether the observed color changes coincide with new vibrational signatures consistent with cresol–metal association (Figs. 2 and 3). Aging with divalent chloride salts produced an isomer-dependent IR response. m-Cresol reproducibly developed a new band at 815 cm^−1^ in the presence of CuCl_2_, FeCl_2_, and ZnCl_2_ (and also with FeSO_4_), whereas p-cresol developed a distinct new band at 775 cm^−1^ only with CuCl_2_ and FeCl_2_ (Table 3). Notably, the emergence of these new bands was not accompanied by a proportional decrease in the intensity of the characteristic cresol bands. This behavior is consistent with the multiphase nature of the system, in which cresol partitioning into droplets and partial metal association occur simultaneously, and with the non-quantitative nature of ATR-FTIR measurements in these heterogeneous samples. No corresponding new band was observed for p-cresol with ZnCl_2_ or FeSO_4_ under identical aging and measurement conditions. Together, these results indicate that formation of the IR-active cresol–metal species depends on both the cresol substitution pattern and the metal/counter-ion environment.

Because these systems are multiphase, mildly acidic, and in some cases chemically evolving during aging, the detailed aqueous speciation of Fe, Cu, and Zn is not uniquely resolved in the present study and is therefore treated qualitatively, with FTIR trends and visual observations used to infer relative differences in metal–cresol association across salts and counter-ions. Mechanistic implications are discussed in the subsections below.

**Table 2:** Visual appearance of separated m- and p-cresol droplets and of the aqueous-rich phase in systems containing 2 wt% cresols and 0.5 M aqueous salt solutions after 10 days

Salt type	Salt in water (no cresols)	Aqueous phase with m- or *p*-cresol	m- or *p*-cresol droplets
NaCl	Transparent	Transparent	Yellow
ZnCl_2_			
ZnSO_4_			
Na_2_SO_4_ (*)			
FeCl_2_	Yellow	Purple	Dark brown
FeSO_4_	Yellow	Yellow	Dark brown
CuCl_2_	Blue	Green	Dark brown
CuSO_4_	Blue	Blue	Yellow

Droplets and color changes were typically visible within approximately 2–24 h, with color becoming more pronounced over time. (*) Samples prepared with Na_2_SO_4_ were monitored for 48 h; within this period, no color change was observed, whereas systems containing FeCl_2_ or CuCl_2_ already exhibited visible droplet discoloration

**Table 3:** Appearance of new IR absorbance bands in m- and p-cresol aqueous 0.5 M salt solutions, after aging for 10 days

Salt type	m-cresol	*p*-cresol
NaCl	No	No
ZnCl_2_	815 cm^−1^	No
ZnSO_4_	No	No
FeCl_2_	815 cm^−1^	775 cm^−1^
FeSO_4_	815 cm^−1^	No
CuCl_2_	815 cm^−1^	775 cm^−1^
CuSO_4_	No	No

#### Interactions of cresols with iron salts

In systems prepared with FeCl_2_, new ATR-FTIR bands appeared in mixtures containing either m-cresol or p-cresol (Table 3; Figs. 2 and 3), coincident with pronounced color changes in both the water-rich phase and the cresol-rich droplets (Table 2). A useful point of comparison is the well-known ferric chloride test for phenols: neutral FeCl_2_ forms intensely colored complexes with phenolic substrates (often violet, blue, or green), commonly attributed to coordination of Fe(III) by phenoxide oxygen to give Fe–OAr species (e.g., Fe(OAr)_2_ and related phenolate-rich complexes) (Eq. 10) [31].

5 $$3ArOH+FeCl_3 \rightarrow Fe(OAr)_3+3HCl$$

Because our experiments start from Fe(II) (FeCl_2_ or FeSO_4_), formation of Fe(III)-phenolate species requires oxidation of Fe^2+^ to Fe^2+^. Since the FeCl_2_ and FeSO_4_ systems were prepared and aged under ambient laboratory atmosphere rather than under inert gas, we cannot assume that iron remained exclusively as dissolved Fe(II) throughout the experiments. Partial oxidation to Fe(III), followed by hydrolysis and formation of iron (oxyhydr)oxide-containing flocs, is therefore plausible under the conditions studied and is consistent with the observed color changes, turbidity, and floc aggregation in the iron-containing samples. Accordingly, the iron results are interpreted here as reflecting chemically evolving Fe-bearing systems rather than strictly Fe(II)-only coordination chemistry. These systems are therefore interpreted at a first-order, phenomenological level rather than through a full coupled redox-speciation model.

Qualitative pH measurements made at the start of the experiments and at selected aging times showed that the salt-containing systems remained mildly acidic overall, with no large bulk pH shifts over 5–10 days. Thus, although Fe(II) oxidation and hydrolysis likely occurred under ambient conditions, these processes were not accompanied by large changes in bulk pH over the experimental timescale.

Oxidation by dissolved oxygen is well established and can be represented as [32]:

6 $$4 Fe ^ {2+}+O_2+4 H^+ \rightarrow 4 Fe^{3+} +2 H_2 O$$

Once Fe(III) is generated, two coupled pathways can proceed. First, complexation of Fe(III) with cresol/cresolate can produce the characteristic purple/violet hues observed in FeCl₂ systems (Table 2), consistent with Fe(III)-phenolate formation. Second, Fe(III) can act as a one-electron oxidant toward phenolic substrates, generating phenoxyl radicals and regenerating Fe(II); subsequent radical coupling can yield dimers and higher-order oligomers/polymers that appear as dark-brown oils or resins. This oxidative-coupling framework is well documented for phenols, and Pummerer’s ketone is a classic product of oxidative coupling of p-cresol-derived phenoxyl radicals [33, 34]. In practical systems, the appearance of dark-brown coloration is often consistent with a mixture of coupled organic products together with iron hydrolysis/precipitation byproducts, rather than with a single pure compound. Consistent with this interpretation, ATR-FTIR comparison of the liquid phase and the precipitate formed in FeCl_2_ and FeSO_4_ systems indicates that the precipitate is enriched in cresol relative to the aqueous phase (Additional file 1: Fig. S15).

The time dependence and salt dependence of the observed colors are also consistent with evolving Fe redox speciation and hydrolysis. In FeCl_2_ solutions, the water-rich phase can develop a purple hue after aging (Table 2), which is consistent with a time window in which sufficient Fe(III) is present to form Fe(III)-phenolate chromophores before extensive hydrolysis, precipitation, and associated changes in aqueous speciation suppress the visible color. In this study, the purple hue was still present after 10 days, although the discoloration became progressively lighter with time (Additional file 1: Fig. S9). Here, the term “acidification” is used descriptively: Fe(III) hydrolysis and associated aqueous equilibria can modify pH and promote formation of iron (oxyhydr)oxide or basic salt phases that both remove Fe(III) from solution and obscure coloration.

In contrast, FeSO_4_ systems did not develop the same purple water-phase hue (Table 2), yet m-cresol still developed a new band at 815 cm^−1^, whereas p-cresol did not (Table 3; Fig. 2). Relative to chloride systems, sulfate can strongly influence Fe(III) aqueous speciation and hydrolysis pathways, promoting formation of Fe(III) oxyhydroxysulfate/basic iron sulfate phases (e.g., sulfate-bearing ferrihydrite or schwertmannite and, under more acidic conditions, jarosite-group phases), which can deplete soluble Fe(III) chromophores and obscure bulk-solution color changes even when Fe-cresol association persists at interfaces and is detected by FTIR [35–37].

Finally, the contrasting FeSO_4_ response of the two isomers suggests that cresol substitution pattern affects the ability to form, or stabilize, IR-detectable Fe–O association under sulfate-influenced speciation. At this stage, we treat the 815 cm⁻¹ (m-cresol) and 775 cm^−1^ (p-cresol) bands as empirical markers of salt- and isomer-dependent interactions (Table 3), rather than assigning them uniquely to a single, universal metal-phenolate structure. A more specific vibrational assignment is developed below by comparing trends across Fe, Cu, and Zn systems and across chloride versus sulfate counter-ions.

#### Interactions of cresols with copper salts

Copper–phenol interactions are well documented and can measurably change copper speciation and reactivity in aqueous systems. Consistent with this, phenolic ligands have been shown to complex Cu^2+^ and reduce its biological availability—for example, Kim et al. reported decreased Cu^2+^ toxicity to Daphnia magna in the presence of phenol [38]. At the molecular level, Mukherjee et al. reported formation of a square-planar Cu(II) complex noncovalently conjugated with p-cresol [9], highlighting that cresol can participate in Cu(II) coordination environments even when the interaction is not purely covalent. These precedents motivate using ATR-FTIR here to test whether cresols form distinct Cu-associated species under the chloride and sulfate electrolyte conditions relevant to our demixing experiments.

In our experiments, ATR-FTIR provides direct evidence for salt-dependent cresol–copper interactions. New bands appeared in systems containing CuCl₂ with either m-cresol or p-cresol (Table 3; Figs. 2D and 3B), whereas no corresponding new bands were observed with CuSO₄ for either isomer under identical aging and measurement conditions (Table 3). This behavior contrasts with the FeSO_4_ case, where m-cresol (but not p-cresol) developed a new band at 815 cm^−1^. Together, the FTIR results reveal a clear hierarchy in which coordination/association is strongly modulated by counter-ion identity and, for some metals, by cresol substitution pattern: CuCl_2_ and FeCl_2_ yield IR-detectable interactions for both isomers, whereas sulfate salts suppress IR-detectable interactions for Cu^2+^ and produce isomer selectivity for Fe^2+^/Fe(III).

The chloride–sulfate contrast is qualitatively consistent with differences in counter-ion-dependent hydration, ion pairing, and ligand exchange behavior in aqueous Cu(II) systems. However, the present data do not uniquely identify the dominant dissolved copper species under these conditions, and the following discussion is therefore intended as a mechanistic interpretation rather than an explicit speciation assignment.

Chloride is generally more labile in the first coordination sphere of divalent transition metals than sulfate, and chloride media more readily permit exchange of aqua ligands by phenolic oxygen donors. In sulfate media, ion pairing and sulfate-influenced hydration can stabilize metal–aqua environments and reduce the propensity for cresol to enter the first coordination sphere, thereby suppressing formation of IR-distinct species. In the specific case of Cu^2+^, this suppression may be amplified by the comparatively strong hydration of Cu^2+^ and its well-known Jahn–Teller distortion (d′), which yields an axially elongated octahedral environment that can be stabilized by solvent and counter-ion structure in solution. Such stabilization can increase the energetic penalty for replacing bound water with a bulky aromatic ligand under the conditions tested.

The observation that m-cresol forms an IR-detectable species with FeSO_4_ but not with CuSO_4_ suggests that the balance between (i) metal–ligand affinity and (ii) sulfate-stabilized hydration/speciation differs between Fe and Cu systems. A parsimonious interpretation consistent with our data is that, in sulfate media, the iron system retains an accessible pathway to form a droplet-associated or interfacial Fe–O(cresol) interaction for the meta isomer (815 cm^−1^), whereas the copper system does not form an analogous IR-distinct association with either isomer within the experimental window. We emphasize that the FTIR results establish the presence or absence of new bands and isomer/counter-ion trends. The detailed coordination geometry, stoichiometry, and identity of the dominant aqueous copper species are therefore treated as mechanistic inferences rather than as features uniquely resolved by FTIR alone.

In summary, CuCl_2_ yields IR-detectable cresol–copper interactions for both m- and p-cresol, whereas CuSO_4_ does not under the same conditions. In combination with the FeCl_2_/FeSO_4_ trends, these results show that sulfate salts can suppress or redirect cresol–metal association and that isomeric structure becomes decisive under sulfate-influenced speciation.

#### Interactions of cresols with zinc salts

In systems prepared with Zn(II) salts, we did not observe the strong visible chromophores seen in the iron systems (Table 2). This is consistent with the electronic structure of Zn^2+^ (d^10^): because the d manifold is filled, Zn(II) complexes are frequently “optically silent” in the visible region, and their photophysics is typically dominated by ligand-centered transitions rather than low-energy metal-centered/charge-transfer transitions that produce intense visible coloration [39]. In addition, Zn(II) is effectively redox-inert under the aqueous conditions relevant here (Zn^2+^/Zn⁰ has E° ≈ −0.76 V), so Zn salts are not expected to participate in Fe-like redox cycling that generates phenoxyl radicals and drives oxidative coupling to brown oligomeric products. Consistent with this expectation, we did not observe the strong brown-oil formation characteristic of the iron-containing systems.

Despite the absence of visible color changes, ATR-FTIR revealed a clear anion- and isomer-dependence for zinc: a new band at 815 cm^−1^ appeared for m-cresol in the presence of ZnCl₂, whereas no corresponding new band was observed with ZnSO_4_ for either isomer under identical aging and measurement conditions (Table 3; Fig. 2A). This indicates that Zn–cresol association can occur without producing visible chromophores and that chloride media favor formation of an IR-distinct species for the meta isomer. At present, however, the detailed aqueous speciation responsible for the ZnCl_2_ versus ZnSO_4_ contrast cannot be uniquely assigned from the FTIR data alone. We therefore interpret this contrast conservatively as evidence that counter-ion-dependent solution chemistry influences whether Zn–cresol association becomes spectroscopically apparent under the conditions tested, while deferring detailed mechanistic assignment pending dedicated speciation calculations.

Finally, the 815 cm^−1^ region falls within the aromatic out-of-plane C–H bending window (≈ 650–1000 cm^−1^) that is well known to be sensitive to substitution pattern and to perturbations of ring electron density. We therefore treat the 815 cm^−1^ band as an empirical marker of m-cresol-specific association in chloride media, while deferring a more detailed vibrational assignment (and its relationship to the Fe and Cu systems) to the cross-salt comparison in the following section(s).

In summary, Zn(II) does not generate the diagnostic visible chromophores or redox-driven coupling observed for iron, but ZnCl₂ nevertheless produces an IR-detectable interaction with m-cresol (815 cm^−1^), whereas ZnSO_4_ does not—highlighting that counter-ion-dependent solution chemistry can govern whether cresol–metal association becomes spectroscopically apparent under the conditions tested [40].

### Environmental implications of droplet formation and floatation behaviour: effect on pollutant transport

#### Floating and sinking behaviour of separated cresol droplets

A key objective of this study was to compare the observed buoyant behavior of cresol-rich droplets against density-based predictions derived from an idealized “perfect separation” model. Qualitative observations of droplet flotation/sinking as a function of salt type and concentration are summarized in Table 4. In several systems, the observed behavior deviated from density-only predictions, most notably in mixtures where ATR-FTIR indicates salt-dependent cresol-metal association (Sect.  3.2) and/or where iron (oxyhydr)oxide flocs formed and visibly entrained droplets.

**Table 4:** Qualitative flotation/sinking behavior of cresol-rich droplets in turbid mixtures aged 10 days (2 wt% cresol; salt concentrations as indicated). Where present, “entrained with flocs” denotes droplet-floc aggregates at the vessel bottom

Salt type	0.1 M	0.5 M
NaCl	N/A	Droplets floated
ZnCl_2_	Some droplets floated, others were entrained with flocs at the bottom	
ZnSO_4_		
CuCl_2_	All droplets sank	Droplets sank
CuSO_4_	Some droplets sank, others floated	Droplets floated
FeCl_2_	Some droplets floated, others were entrained with flocs at the bottom	
FeSO_4_		

Buoyancy calculations are used here only as a first-order conceptual benchmark to distinguish conditions under which salt-induced cresol-rich droplets would be expected to rise or sink based on density contrast alone; they are not intended as predictive models of droplet terminal velocity or multiphase transport in real aquifers. To establish this baseline, calculations assumed: (i) perfect phase separation between water and cresol, such that the droplet phase has the density of neat m- or p-cresol, and (ii) no droplet entrainment by metal (oxyhydr)oxide flocs.

Under these assumptions, buoyancy is governed by the density contrast between cresol (ρ(m-cresol) ≈ 1.034 g mL^−1^; ρ(p-cresol) ≈ 1.0341 g mL^−1^) and the salt solution at 20 °C. Salt-solution densities were estimated using Laliberté and Cooper [17]. The corresponding switch concentrations at which the brine becomes denser than cresol, and droplets should therefore transition from sinking to floating under the perfect-separation assumption, are summarized in Table 5. The associated density-only Stokes predictions are shown in Additional file 1: Figs. S17–S19, which illustrate the same sign change across representative droplet sizes.

Using density alone, the perfect-separation model predicts that 0.1 M brines are generally not dense enough to buoy cresol, whereas most of the divalent salts at 0.5 M should be. For NaCl, the predicted threshold is approximately 0.90 m (mol kg^−1^ H_2_O), substantially above the concentrations examined here. For the divalent salts, the predicted switch concentrations are lower (approximately 0.22–0.33 m depending on salt; Table 5), implying that at 0.5 M droplets should typically float if they retain near-neat cresol density and if no other processes modify their effective density or mobility.

**Table 5:** Predicted switch concentration (density-only) at which the brine density equals the cresol density ($\:{\rho\:}_{brine}={\rho\:}_{cresol}$), implying a transition from sinking to floating under the perfect-separation assumption (droplet density = neat cresol) and neglecting droplet-floc entrainment. Switch concentrations are reported as molality (* m*, mol kg^−1^ H_2_O) for direct comparison to density correlations; experimentally prepared solutions are described by molarity (M). At 20 °C, these switch molalities correspond approximately to $\:\sim\:$0.22–0.33 M for the divalent salts and $\:\sim\:$0.9 M for NaCl. Differences between m- and p-cresol thresholds are negligible ($\:\le\:$0.003 m) because their densities are nearly identical

Salt	Switch molality m (mol/kg H_2_O) (m-cresol)	Switch molality m (mol/kg H_2_O) (*p*-cresol)
NaCl	0.903	0.906
CuCl_2_	0.295	0.296
CuSO_4_	0.224	0.225
ZnCl_2_	0.289	0.290
ZnSO_4_	0.219	0.220
FeCl_2_	0.330	0.331
FeSO_4_	0.237	0.238

We nevertheless observed systematic departures from these idealized predictions. First, salting out does not necessarily yield a neat-cresol droplet: retention of water and/or dissolved salts within the cresol-rich phase can shift droplet density toward that of the surrounding aqueous phase, thereby weakening the buoyancy contrast relative to the perfect-separation assumption. Second, where ATR-FTIR indicates cresol-metal association, incorporation of metal-bearing species into the droplet phase may further alter effective droplet density and interfacial behavior. Third, in Fe-containing systems, iron (oxyhydr)oxide formation produced visible flocs that directly entrained droplets (Fig. 1), so the settling behavior of droplet-floc aggregates cannot be compared directly with density-only predictions for isolated liquid droplets.

These buoyancy-controlled phase-partitioning outcomes have direct implications for cresol transport and persistence in saline- and metal-impacted aquifers. Floating droplets can accumulate beneath the water table or under low-permeability layers, where they may act as persistent secondary sources that slowly repartition cresol back into the dissolved phase. Conversely, sinking droplets, or droplet-floc aggregates, can migrate downward into lower-permeability intervals, increasing the likelihood of retention by straining, longer residence times, and later back-diffusion into mobile porewater. In both cases, conversion of dissolved cresol into a dispersed droplet phase shifts transport away from purely solute advection-dispersion toward a coupled multiphase/colloid-like regime in which droplet size distribution, density contrast, and attachment/straining processes become controlling parameters. This transition also motivates filtration-based removal strategies (Sect.  3.4), because droplets and droplet-floc aggregates behave as separable particulate phases rather than purely dissolved solute.

#### Effect of salts on the diffusion of m-cresol and p-cresol in groundwater

The diffusion calculations presented in this section should be interpreted as order-of-magnitude scaling arguments that illustrate how salt-induced phase separation shifts cresol transport from molecular diffusion toward particle-like mobility as entity size increases, rather than as full predictive descriptions of subsurface transport. We first consider diffusion of dissolved m-cresol/p-cresol at concentrations below the aqueous solubility limit, in the absence of added salts. For small organic molecules in water near 20 °C, molecular diffusion coefficients are typically on the order of 0.5–1.0 × 10^−3^ m^2^ s^−1^, and cresols (MW ≈ 108 g mol^−1^) fall within this range. A practical estimate for both m-cresol and p-cresol in water at 20 °C is therefore $D_{aq,dissolved} \approx (0.6- 0.9) {\times}10^{-9} m^{2} s^{-1}$. For subsequent estimates we use $D_{aq,dissolved} \approx 0.8 {\times} 10^{-9} m^{2} s^{-1}$.    

As discussed in the materials and methods section, the characteristic diffusive length scale is:

$$\:\mathcal{l}\sim\:\sqrt{2\mathrm{D}\mathrm{t}}$$

which yields, for $\:\mathrm{D}=0.8\times\:{10}^{-9}$ $m^2 s^{-1}$ and $\:\mathcal{l}\approx\:2.4\:\:\mathrm{m}\mathrm{m}$ over 1 h and $\:\mathcal{l}\approx\:12\:\mathrm{m}\mathrm{m}$ over 1 d. In most porous media, advection dominates transport in the mobile pore space unless groundwater velocities are extremely small. However, diffusion becomes central when contaminants exchange mass with low-permeability domains, such as rock matrices adjacent to fractures or fine-grained aquitards. In such settings, “matrix diffusion” creates a large, slowly exchanging storage reservoir: during source loading, solutes advect along transmissive pathways while simultaneously diffusing into the adjacent stagnant porewater; after source control or remediation, concentration gradients reverse and contaminants diffuse back, producing persistent tailing and plume longevity over years to decades [41, 42].

When salts induce droplet formation (or sub-micron clustering), the relevant diffusivity is no longer molecular diffusion but Brownian (particle) diffusion, which can be estimated by the Stokes–Einstein relation (Eq. 6):

$$\:\begin{array}{cccc}&\:{\mathrm{D}}_{\mathrm{p}}=\frac{{\mathrm{k}}_{\mathrm{B}}\mathrm{T}}{6{\uppi\:}{\upmu\:}\mathrm{a}}&\:&\:\end{array}$$

where $\:\mathrm{a}$ is particle radius, $\:{\upmu\:}$ is dynamic viscosity, $\:\mathrm{T}$ is temperature, and $\:{\mathrm{k}}_{\mathrm{B}}$ is the Boltzmann constant. Table 6 summarizes predicted particle diffusion coefficients at 20 °C assuming $\:{\upmu\:}$ equals that of water. Optical microscopy indicates that droplets were polydisperse, with observed diameters spanning 1–50 μm, and droplets as large as 0.5 mm were seen with the naked eye; only droplets larger than ~ 1 μm are resolvable by optical microscopy, but sub-micron droplets cannot be excluded. Sub-micron droplets have been reported for other water-miscible solvents desolvated by salts [20, 43–46], and, if present here, would exhibit comparatively rapid Brownian spreading (centimeters to decimeters over hour timescales in still water). In contrast, droplets ≥ 10 μm diffuse only millimeters to centimeters per hour and, under most groundwater flow conditions, behave primarily as advected particles. For droplets ≥ 50 μm, buoyancy/settling (Sect. 3.3.1) becomes increasingly important relative to Brownian diffusion. Given the observed polydispersity and the plausible presence of sub-micron droplets, salts are expected to generate a broad distribution of effective diffusivities for cresol in groundwater.

**Table 6:** Effect of droplet size on predicted diffusion coefficients for cresol-rich droplets at 20 °C, estimated using Stokes–Einstein and assuming $\:{\upmu\:}={{\upmu\:}}_{\mathrm{w}\mathrm{a}\mathrm{t}\mathrm{e}\mathrm{r}}$

Droplet diameter $\:\mathrm{d}$	radius $\:\mathrm{a}$	$\:{\mathrm{D}}_{\mathrm{p}}$(m²/s)	Diffusion distance in 1 h $\:\sqrt{2\mathrm{D}\mathrm{t}}$
0.1 μm	5e^− 8^ m	~ 4.2e–6	~ 0.17 m
1 μm	5e^− 7^ m	~ 4.2e–7	~ 0.055 m
10 μm	5e^− 6^ m	~ 4.2e–8	~ 0.017 m
50 μm	2.5e^− 5^ m	~ 8.4e–9	~ 0.0078 m
0.5 mm	2.5e^− 4^ m	~ 8.4e–10	~ 0.0025 m

Finally, metal association (Sect.  3.2) can further modify mass transport beyond salting out alone. Salting out can increase the bulk viscosity of the aqueous phase, which reduces diffusion coefficients for all dissolved species (and reduces Brownian diffusivity via Stokes–Einstein). In addition, cresol–metal association can shift cresol mass into larger, more slowly diffusing entities—for example, charged complexes, ion-paired species, or early-stage clusters that precede visible droplets. Such growth in effective hydrodynamic size naturally reduces diffusivity (Eq. 6) and can delay redistribution of cresol between domains (e.g., between mobile porewater and low-permeability zones, or between the aqueous phase and separable particulate phases). We therefore expect that systems showing IR evidence of cresol–metal association will exhibit slower effective diffusion/redistribution than salting-out-only systems, even before macroscopic droplets become apparent.

#### Effect of salting out on straining

The straining analysis presented in this section is intended as a first-order screening tool to identify when droplet size may become large enough for pore-throat exclusion to matter, not as a predictive retention model for natural aquifers with heterogeneous pore geometry and coupled physicochemical interactions.

Salting out converts dissolved cresol into droplets and clusters whose mobility can become limited by geometric straining in porous media. Using the geometric framework described in Sect. 2.8, a characteristic pore-throat radius is estimated as $r_t \approx \:{\upalpha\:} d_g \:{\upepsilon\:}/(1-\:{\upepsilon\:})$ with $\:{\upalpha\:}=0.1$, and droplets are considered likely to be strained when $\:{\mathrm{d}}_{\mathrm{p}}\gtrsim\:2{\mathrm{r}}_{\mathrm{t}}$ (i.e., when droplet diameter exceeds the characteristic throat diameter) [11, 12].

At a fixed grain size $\:{\mathrm{d}}_{\mathrm{g}}$, increasing porosity $\:{\upepsilon\:}$ increases $\:{\mathrm{r}}_{\mathrm{t}}$; higher-porosity media therefore impose weaker geometric exclusion and can transmit larger droplets before straining becomes significant. Applying the criterion shows that the droplet sizes most susceptible to pore-throat exclusion depend strongly on soil texture (grain size) and porosity. For coarse sand $\:{\mathrm{d}}_{\mathrm{g}}$= 500 μm, $\:{\upepsilon\:}=0.35$), $\:{\mathrm{r}}_{\mathrm{t}}\approx\:$27 μm, corresponding to a straining threshold of $\:{\mathrm{d}}_{\mathrm{p}}\gtrsim\:$54 μm. Under these conditions, droplets in the 1–10 μm range would be expected to pass geometric constrictions, whereas droplets approaching tens of microns may begin to experience partial screening. For medium sand $\:{\mathrm{d}}_{\mathrm{g}}$= 250 μm, $\:{\upepsilon\:}=0.35$), $\:{\mathrm{r}}_{\mathrm{t}}\approx\:$13.5 μm and $\:{\mathrm{d}}_{\mathrm{p}}\gtrsim\:$27 μm, shifting the cutoff into the upper end of the droplet size range observed experimentally. In finer media, predicted throat sizes decrease sharply: for silt $\:{\mathrm{d}}_{\mathrm{g}}$= 50 μm, $\:{\upepsilon\:}=0.35$), $\:{\mathrm{r}}_{\mathrm{t}}\approx\:$4.1 μm and straining is expected for droplets $\:{\mathrm{d}}_{\mathrm{p}}\gtrsim\:$8 μm; for fine silt/clay-sized aggregates $\:{\mathrm{d}}_{\mathrm{g}}$= 10 μm, $\:{\upepsilon\:}=0.5$), $\:{\mathrm{r}}_{\mathrm{t}}\approx\:$1 μm and straining is expected for droplets $\:{\mathrm{d}}_{\mathrm{p}}\gtrsim\:$2 μm.

Collectively, these estimates imply that sub-micron clusters would generally evade geometric straining even in fine media (although other retention mechanisms may still operate), whereas multi-micron droplets are increasingly screened as grain size decreases and/or pore space becomes more constricted. This provides a straightforward physical rationale for why, under salt-induced two-phase conditions, larger droplets may be immobilized near the source zone in fine-grained soils, while smaller clusters remain mobile and contribute to longer-range transport.

### Potential impact of salts on water purification approaches

The physical state of cresols in saline and metal-bearing waters—dissolved, phase-separated droplets, and/or metal-associated species—directly affects which removal strategies are likely to be effective. In the present study, optical microscopy showed that salt addition produced cresol-rich droplets in the micron-and-larger size range (Fig. 1; Additional file 1: Figs. S3–S8), while bottle tests further showed that these droplets could float, sink, or become entrained with flocs depending on salt identity (Table 4; Additional file 1: Figs. S9–S14). These observations indicate that once phase separation occurs, physical separation strategies such as skimming, settling, or filtration may become relevant, but the preferred approach depends on whether droplets are buoyant, suspended, or associated with denser aggregates.

In our experiments, p-cresol droplets more consistently exhibited buoyant behavior, whereas systems in which ATR-FTIR indicated cresol-metal association, particularly for m-cresol in chloride and some iron-containing systems, more often produced droplets that settled, became entrained with flocs, or remained suspended in the water column. This divergence suggests that depending on the salt environment, surface skimming of buoyant droplets, settling of denser droplets or droplet-floc aggregates, filtration, or a combination of these approaches may be preferable.

Filtration also provided a useful removal strategy for cresol solutions in the absence of salts. Nylon filters removed 86 ± 13% of m-cresol and 82 ± 10% of p-cresol from 2 wt% cresol solutions, whereas MCE filters removed 39 ± 10% and 43 ± 3% , respectively. Because the two filters had comparable nominal pore sizes but different removal efficiencies, the results indicate that filter material and surface properties influence cresol removal in addition to any simple size-screening effect. Although optical microscopy showed droplets in the micron-and-larger range, we did not determine the full droplet-size distribution by light scattering or related methods, and therefore do not interpret the filtration results as arising solely from pore-size exclusion.

The performance of filtration could be improved by adding polyvinylpyrrolidone (PVP). PVP is a benign polymer widely used to bind polyphenols via hydrogen bonding between the PVP carbonyl group and phenolic OH groups [32–35]. For example, Fan et al. reported hydrogen bonding between PVP carbonyl groups and phenolic OH groups in tannic acid-PVP systems [36]. Upon addition of 1 wt% PVP, MCE filters removed 89 ± 2% of m-cresol and 73 ± 6% of p-cresol. This result is consistent with polymer-mediated conversion of dissolved cresols into larger and/or more strongly retained species and suggests that combining binding with filtration can extend treatment performance from salt-induced two-phase systems to salt-free waters.

PVP was also tested in mixtures containing 2 wt% p-cresol and 0.5 M FeCl_2_, FeSO_4_, CuCl_2_, or CuSO_4_. In these systems, the polymer visibly aggregated droplets, and after centrifugation, ATR-FTIR comparison of the solids and supernatant showed that the solids were cresol-rich relative to the aqueous phase (Additional file 1: Fig. S16). Together, these results support a treatment framework in which phase separation, aggregation/binding, and filtration or settling can be combined, with the most effective sequence depending on whether cresol is present predominantly as dissolved solute, buoyant droplets, or denser droplet-associated aggregates.

## Conclusions

This study shows that electrolyte composition can fundamentally change cresol speciation in water—from a primarily dissolved solute to a dispersed cresol-rich droplet phase—and that transition metals can further modify the separated phase through salt- and isomer-dependent association. Bottle tests and optical microscopy demonstrate that 0.1–0.5 M chloride and sulfate salts reduce the miscibility of m-cresol and p-cresol and generate polydisperse droplets. COSMO-RS calculations support this salting-out behavior by predicting large increases in cresol activity in saline water. Beyond demixing, ATR-FTIR reveals diagnostic, isomer-dependent new bands (815 cm⁻¹ for m-cresol and 775 cm⁻¹ for p-cresol) in selected metal-salt systems, indicating that cresol–metal association depends strongly on both cresol substitution pattern and the metal/counter-ion environment; chloride media consistently favor IR-distinct interactions relative to sulfate media. Density-only “perfect separation” predictions indicate that divalent brines at sufficiently high concentration should buoy cresol droplets, yet observed flotation and settling deviate systematically where metal association and iron (oxyhydr)oxide floc formation increase effective droplet density and promote entrainment. These coupled effects imply that cresol migration in impacted aquifers can shift from classical solute advection–dispersion to a multiphase/particle-like regime governed by droplet size distribution, buoyancy, aggregation with mineral flocs, and pore-throat straining. Finally, the work discusses the impact of the phase behaviour of m- and p-cresol on possible water purification approaches, which can include skimming or settling depending on the buoyancy of the droplets, filtration and use of benign additives such as PVP. Overall, the transport calculations presented here should be viewed as conceptual/scaling analyses that identify plausible transport regimes and controlling mechanisms following salt-induced cresol demixing. Resolving site-specific plume behavior would require full multiphase reactive transport treatment beyond the scope of the present study.

## Supplementary Information

Below is the link to the electronic supplementary material.

**Additional file 1**.

## Data Availability

Data will be made available upon reasonable request to the corresponding author.
