# Supplementary material for: Cresol salting out and complexation with metal salts influence their migration in impacted aquifers
Source: Discov Water. 2026 Jun 16;6(1):95. doi: 10.1007/s43832-026-00402-6 (PMC13506607; doi:10.1007/s43832-026-00402-6)
Supplement: Supplementary file 1 [file 43832_2026_402_MOESM1_ESM.docx]

**Supporting Information**

**Cresol Salting Out and Complexation with Metal Salts Influence their Migration in Impacted Aquifers**

Renato Souza Lima Sant’Anna^1^, Alejandro G. Marangoni^2,3^, Erica Pensini^1,2*^

^1^ Department of Civil, Environmental and Water Engineering, College of Engineering, University of Guelph, 50 Stone Road East, Guelph (ON), N1G 2W1, Canada

^2^Biophysics Interdepartmental Group (BIG), University of Guelph, 50 Stone Road East, Guelph (ON), N1G 2W1, Canada

^3^Food Science Department, University of Guelph, 50 Stone Road East, Guelph (ON), N1G 2W1, Canada

*Corresponding author: email: epensini@uoguelph.ca; phone: +1 519-824-4120 ext. 56746

**Section S1 Calibration curve used for m-cresol and p-cresol quantification using ATR-FTIR**

**Figure S1** Calibration curve used for m-cresol and p-cresol quantification using ATR-FTIR.

**Section S2 Parameters used in COSMO-RS calculations**

**Figure S2** Parameters used in COSMO-RS calculations.

**Section S3 Density-model implementation for flotation calculations**

To estimate buoyancy-driven behavior after phase separation, the density of the aqueous electrolyte solution, $\rho_{\mathrm{brine}}$, was computed using the Laliberté–Cooper semi-empirical density model for single-electrolyte aqueous solutions [17], assuming complete phase separation between a pure cresol droplet phase and the surrounding aqueous electrolyte phase.

At a given temperature $t$(°C), the density of pure water $\rho_{w}$(kg m^-3^) was calculated as

$\begin{matrix} & \rho_{w}(t)=\frac{999.83952+16.945176\text{ }t-0.0079870401\text{ }t^{2}-4.6170461\times{10}^{-5}\text{ }t^{3}+1.0556302\times{10}^{-7}\text{ }t^{4}-2.8054253\times{10}^{-10}\text{ }t^{5}}{1+0.01687985\text{ }t} & & \end{matrix}$	(S1)

and the corresponding specific volume was obtained as

$\begin{matrix} & \bar{v}_{w}(t)=\frac{1}{\rho_{w}(t)} & & \end{matrix}$	(S2)

For an electrolyte $i$, the apparent specific volume $\bar{v}_{\mathrm{app},i}$(m^3^ kg^-1^) was computed from the electrolyte mass fraction $w_{i}$ using

$\begin{matrix} & \bar{v}_{\mathrm{app},i}(w_{i},t)=\frac{w_{i}+c_{2}+c_{3}t}{(c_{0}w_{i}+c_{1})\exp\left( {10}^{-6}(t+c_{4})^{2} \right)} & & \end{matrix}$	(S3)

where $c_{0}$–$c_{4}$ are electrolyte-specific constants.

The brine density was then obtained from the mixing rule

$\begin{matrix} & \rho_{\mathrm{brine}}=\frac{1}{(1-w_{i})\bar{v}_{w}+w_{i}\bar{v}_{\mathrm{app},i}} & & \end{matrix}$	(S4)

To evaluate $\rho_{\mathrm{brine}}$ at a target molarity $M$(mol L^-1^), the model was implemented on a 1 kg-water basis using the auxiliary molality $m$(mol kg^-1^). The electrolyte mass fraction was expressed as

$\begin{matrix} & w_{i}=\frac{mM_{W}}{1+mM_{W}} & & \end{matrix}$	(S5)

where $M_{W}$ is the electrolyte molar mass in kg mol^-1^. The corresponding molarity was calculated as

$\begin{matrix} & M(m)=\frac{m\text{ }\rho_{\mathrm{brine}}}{1000(1+mM_{W})} & & \end{matrix}$	(S6)

and $m$ was solved numerically such that $M(m)=M_{\mathrm{target}}$. The resulting $w_{i}$ was then used to evaluate $\rho_{\mathrm{brine}}$.

The limiting salt concentration for flotation, $M^{*}$, was defined by the condition

$\begin{matrix} & \rho_{\mathrm{brine}}(M^{*})=\rho_{\mathrm{drop}} & & \end{matrix}$	(S7)

where $\rho_{\mathrm{drop}}$ is the density of pure cresol. As described in the main text, rise/sink velocities were then estimated using Stokes’ law.

**Section S4 Assumptions used for diffusion coefficient estimates**

Diffusion calculations were intended as first-order scaling estimates to compare likely transport regimes for dissolved cresol versus cresol-rich droplets/clusters after phase separation, rather than as predictive models of aquifer-scale transport.

All estimates were performed at 20 °C (T=293.15 K) using water as the continuous phase, with dynamic viscosity taken as $\mu$ =1.002∙10^-3^ Pa∙s. For dissolved transport below the solubility limit, the aqueous diffusion coefficient of cresol was assigned a representative value of

D_aq_≈0.8∙10^-9^ m^2^∙s^-1^	(S8)

for both m-cresol and p-cresol. This value was selected as a typical room-temperature diffusivity for neutral organic solutes of similar molecular size in water.

For the two-phase case, cresol-rich droplets/clusters were treated as spherical particles with radius $a$, and their Brownian diffusion coefficient was evaluated using Eq. (6) in the main text over the experimentally relevant size range, spanning sub-micron to multi-micron droplets.

Characteristic diffusive spreading lengths were estimated using Eq. (7) in the main text. These estimates were used only to compare the relative magnitudes of dissolved-phase and droplet-phase spreading over time.

**Section S5 Geometric straining model details**

Potential screening of cresol-rich droplets in porous media was evaluated using a first-order geometric straining framework intended only to illustrate likely size-exclusion behavior. The model neglects adhesion/attachment to grains, chemical interactions, capillary effects, droplet deformability, and droplet–floc entrainment.

Droplets were treated as rigid spheres of diameter $d_{p}$ transported through a granular medium characterized by grain diameter $d_{g}$ and porosity $\varepsilon$. A characteristic pore-throat radius $r_{t}$was approximated using the packing-based scaling relation

$\begin{matrix} & r_{t}\approx\alpha d_{g}\frac{\varepsilon}{1-\varepsilon} & & \end{matrix}$	(S9)

where $\alpha$ is a dimensionless geometric factor describing the ratio between grain size and a representative constriction size. For order-of-magnitude estimates, a mid-range value of $\alpha=0.1$ was used.

Under pure straining control, droplets were classified as likely to be strained if their diameter exceeded the characteristic throat diameter,

$\begin{matrix} & d_{p}\gtrsim2r_{t} & & \end{matrix}$	(S10)

whereas droplets with $d_{p}\ll2r_{t}$ were treated as likely to pass geometric constrictions. This framework was used as a conservative first-pass criterion for droplet immobilization by pore-throat exclusion in porous media of different textures and porosities.

**Section S6 Optical microscopy: supporting images**

**Figure S3** Optical microscopy of aqueous solutions containing 2 wt% *p*-cresol and either 0.5 M FeCl_2_ (A, B) or 0.1 M FeCl_2_ (C, D) or 2 wt% *m*-cresol and either 0.5 M FeCl_2_ (E, F) or 0.1 M FeCl_2_ (G, H). The scale bar is 100 µm. Note that the edges of the samples are visible in images B, H.

**Figure S4** Optical microscopy of aqueous solutions containing 2 wt% *p*-cresol and either 0.5 M FeSO_4_ (A, B) or 0.1 M FeSO_4_ (C, D) or 2 wt% *m*-cresol and either 0.5 M FeSO_4_ (E, F) or 0.1 M FeSO_4_ (G, H). The scale bar is 100 µm. Note that the edges of the samples are visible in images B, C, D, E, F and H.

**Figure S5** Optical microscopy of aqueous solutions containing 2 wt% p-cresol and either 0.5 M CuCl_2_ (A, B) or 0.1 M CuCl_2_ (C, D) or 2 wt% m-cresol and either 0.5 M CuCl_2_ (E, F) or 0.1 M CuCl_2_ (G, H). The scale bar is 100 µm. Note that the edges of the samples are visible in images B, C, D, E, G and H.

**Figure S6** Optical microscopy of aqueous solutions containing 2 wt% *p*-cresol and either 0.5 M CuSO_4_ (A, B) or 0.1 M CuSO_4_ (C, D) or 2 wt% *m*-cresol and either 0.5 M CuSO_4_ (E, F) or 0.1 M CuSO_4_ (G, H). The scale bar is 100 µm. Note that the edges of the samples are visible in all images.

**Figure S7** Optical microscopy of aqueous solutions containing 2 wt% *p*-cresol and either 0.5 M ZnCl_2_ (A, B) or 0.1 M ZnCl_2_ (C, D) or 2 wt% *m*-cresol and either 0.5 M ZnCl_2_ (E, F) or 0.1 M ZnCl_2_ (G, H). The scale bar is 100 µm. Note that the edges of the samples are visible in images C, D, F, and G.

**Figure S8** Optical microscopy of aqueous solutions containing 2 wt% *p*-cresol and either 0.5 M ZnSO_4_ (A, B) or 0.1 M ZnSO_4_ (C, D) or 2 wt% *m*-cresol and either 0.5 M ZnSO_4_ (E, F) or 0.1 M ZnSO_4_ (G, H). The scale bar is 100 µm. Note that the edges of the samples are visible in all images, except C.

**Section S7 Bottle tests: supporting images**

**Figure S9** Temporal evolution of vials containing aqueous FeCl_2_ (0.1 M or 0.5 M) solutions with 2 wt% m- and p-Cresol.

**Figure S10** Temporal evolution of vials containing aqueous FeSO_4_ (0.1 M or 0.5 M) solutions with 2 wt% m- and p-Cresol.

**Figure S11** Temporal evolution of vials containing aqueous CuCl_2_ (0.1 M or 0.5 M) solutions with 2 wt% m- and p-Cresol.

**Figure S12** Temporal evolution of vials containing aqueous CuSO_4_ (0.1 M or 0.5 M) solutions with 2 wt% m- and p-Cresol.

**Figure S13** Temporal evolution of vials containing aqueous ZnCl_2_ (0.1 M or 0.5 M) solutions with 2 wt% m- and p-Cresol.

**Figure S14** Temporal evolution of vials containing aqueous ZnSO_4_ (0.1 M or 0.5 M) solutions with 2 wt% m- and p-Cresol.

**Section S8 ATR-FTIR: supporting spectra**

**Figure S15** ATR-FTIR spectra of the liquid phase and the precipitate in samples prepared using 2 wt% m- or p-cresol and 0.5 M FeCl_2_ and FeSO_4_ between 950-1800 cm^-1^. The comparison between the δ(HOH) band of water (≈1600 cm^-1^) and the ν(C=C) ring stretching bands of m-cresol (≈1490 cm^-1^) and p-cresol (≈1515 cm^-1^) qualitatively shows that the precipitate is rich in m- and p-cresol.

**Figure S16** Comparison between absorbance spectra probed by ATR-FTIR of the supernatant and solid deposit formed in samples containing 2 wt% p-cresol, 0.5 M salts: (A) FeCl_2_, (B) FeSO_4_, (C) CuCl_2_ or (D) CuSO_4_; and 1 wt% PVP, after centrifugation.

**Section S9 Terminal Stokes velocity: supplementary graphs**

**Figure S17.** Density-only (perfect-separation) predictions for buoyant rise or settling of cresol-rich 0.5 mm large droplets in aqueous salt solutions at 20 °C. Terminal velocities were calculated using Stokes’ law for a spherical droplet of radius r = 0.5 mm in a fluid of viscosity μ = 1.002 mPa·s, using $\Delta\rho=\rho_{\text{brine}}-\rho_{\text{cresol}}$ with $\rho_{\text{cresol}}$ set to neat m-cresol or p-cresol and $\rho_{\text{brine}}$estimated from Laliberté and Cooper [17]. Results are shown for (A) m-cresol and (B) p-cresol. Positive velocities denote upward motion (brine denser than cresol; droplets float), and negative velocities denote downward motion (droplets sink). Calculations neglect water uptake into droplets and any droplet–floc aggregation/entrainment; thus, deviations from these predictions indicate non-ideal phase separation and/or particle-assisted settling/rising in the experiments.

**Figure S18** Density-only (perfect-separation) predictions for buoyant rise or settling of cresol-rich 1 μm large droplets in aqueous salt solutions at 20 °C. Terminal velocities were calculated using Stokes’ law for a spherical droplet of radius r = 1 μm in a fluid of viscosity μ = 1.002 mPa·s, using $\Delta\rho=\rho_{\text{brine}}-\rho_{\text{cresol}}$ with $\rho_{\text{cresol}}$ set to neat m-cresol or p-cresol and $\rho_{\text{brine}}$ estimated from Laliberté and Cooper [17]. Results are shown for (A) m-cresol and (B) p-cresol. Positive velocities denote upward motion (brine denser than cresol; droplets float), and negative velocities denote downward motion (droplets sink). Calculations neglect water uptake into droplets and any droplet–floc aggregation/entrainment; thus, deviations from these predictions indicate non-ideal phase separation and/or particle-assisted settling/rising in the experiments.

**Figure S19** Density-only (perfect-separation) predictions for terminal Stokes velocity of buoyant rise or settling of 50 μm large cresol-rich droplets in aqueous salt solutions at 20 °C. Terminal velocities were calculated using Stokes’ law for a spherical droplet of radius r = 50 μm in a fluid of viscosity μ = 1.002 mPa·s, using $\Delta\rho=\rho_{\text{brine}}-\rho_{\text{cresol}}$with $\rho_{\text{cresol}}$ set to neat m-cresol or p-cresol and $\rho_{\text{brine}}$ estimated from Laliberté and Cooper [17]. Results are shown for (A) m-cresol and (B) p-cresol. Positive velocities denote upward motion (brine denser than cresol; droplets float), and negative velocities denote downward motion (droplets sink). Calculations neglect water uptake into droplets and any droplet–floc aggregation/entrainment; thus, deviations from these predictions indicate non-ideal phase separation and/or particle-assisted settling/rising in the experiments.
